# Characterization of the Blood Brain Barrier Disruption in the Photothrombotic Stroke Model

**DOI:** 10.3389/fphys.2020.586226

**Published:** 2020-11-12

**Authors:** Rebecca Z. Weber, Lisa Grönnert, Geertje Mulders, Michael A. Maurer, Christian Tackenberg, Martin E. Schwab, Ruslan Rust

**Affiliations:** ^1^Institute for Regenerative Medicine, University of Zurich, Zurich, Switzerland; ^2^Neuroscience Center Zurich, University of Zurich and ETH Zurich, Zurich, Switzerland; ^3^Department of Health Sciences and Technology, ETH Zurich, Zurich, Switzerland

**Keywords:** photothrombotic stroke, BBB, ischemia, leakage, edema, pericytes, tight junctions

## Abstract

Blood brain barrier (BBB) damage is an important pathophysiological feature of ischemic stroke which significantly contributes to development of severe brain injury and therefore is an interesting target for therapeutic intervention. A popular permanent occlusion model to study long term recovery following stroke is the photothrombotic model, which so far has not been anatomically characterized for BBB leakage beyond the acute phase. Here, we observed enhanced BBB permeability over a time course of 3 weeks in peri-infarct and core regions of the ischemic cortex. Slight increases in BBB permeability could also be seen in the contralesional cortex, hippocampus and the cerebellum at different time points, regions where lesion-induced degeneration of pathways is prominent. Severe damage of tight and adherens junctions and loss of pericytes was observed within the peri-infarct region. Overall, the photothrombotic stroke model reproduces a variety of features observed in human stroke and thus, represents a suitable model to study BBB damage and therapeutic approaches interfering with this process.

## Introduction

Stroke is a leading cause of disability and death worldwide. There are over 13.7 million strokes every year and one in four people over age 25 will experience a stroke in their lifetime (GBD 2016 Stroke Collaborators, 2019). A majority of stroke victims cannot profit from thrombolysis or thrombectomy, and rehabilitation therapies have often only limited success in restoring the lost functions. Stroke, therefore, remains a global substantial social and clinical burden (Katan and Luft, 2018).

A hallmark of stroke pathology is the breakdown of the blood brain barrier (BBB) that can persist for up to several weeks after stroke and is associated with poor patient outcomes (Kastrup et al., 2008; Brouns et al., 2011). Anatomically, BBB breakdown is characterized by alterations of tight junction protein complexes and damage to cellular components of the neurovascular unit, e.g., pericytes (Bell et al., 2010; Prakash and Carmichael, 2015; Abdullahi et al., 2018; Nation et al., 2019; Montagne et al., 2020). As a result of enhanced BBB permeability, many secondary injury cascades are activated including cytotoxic edema, a disruption in cellular water and ion homeostasis and vasogenic edema, fluid extravasation into the brain parenchyma (Zhao et al., 2015). BBB integrity is also a safety measure for novel experimental therapies aiming at revascularization of the ischemic brain since newly formed blood vessels can lack barrier functions and may exacerbate brain damage (Rust et al., 2018, 2019c). On the other hand, delivery of therapeutic drugs may be facilitated by the improved accessibility of the brain (Obermeier et al., 2013).

In pre-clinical research different rodent models are used to study various aspects of stroke pathophysiology and to evaluate novel treatment approaches (Carmichael, 2005; Dirnagl, 2010). Apart from the frequently used model of middle cerebral artery occlusion (MCAO), the photothrombotic stroke is an ever-increasingly used model with well-characterized sensory-motor deficits and long-term recovery. However, BBB injury has not yet been studied beyond the acute stage of photothrombotic stroke (Hoff et al., 2005).

Here, we provide a 3-week time course of BBB permeability changes in mice subjected to a cortical photothrombotic stroke within the stroke core and peri-infarct regions. We also assess the permeability in neighboring and contralesional brain regions including contralesional sensorimotor cortex, hippocampus, cerebellum and visual cortex. Moreover, we quantify damage to cellular and major junction protein components in ischemic regions following the injury.

## Results

### Blood Brain Barrier Leakage Is a Hallmark of the Photothrombotic Stroke Model

**FIGURE 1:**
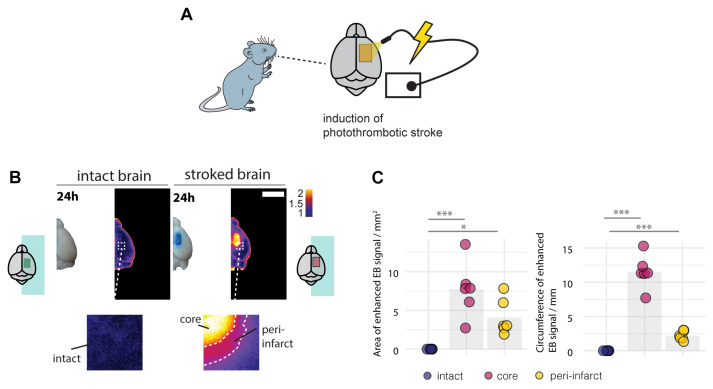
Disruption of the blood brain barrier 24 h following the photothrombotic stroke. **(A)** Schematic representation of photothrombotic stroke induction in the sensorimotor cortex. **(B)** Macroscopic and pseudo-colored image in stroked and non-stroked animals showing the Evans blue (EB) extravasation in stroke core and penumbra. Scale bar: 5 mm. **(C)** Quantitative assessment of area and circumference of EB signals. Each dot in the plots represents one animal and significance of mean differences between the groups was assessed using Tukey’s HSD. Asterisks indicate significance: ^∗^*P* < 0.05, ^∗∗^*P* < 0.01, ^∗∗∗^*P* < 0.001.

Breakdown of the BBB and the risk of hemorrhagic transformation usually occurs within the first days in stroke patients (Nadareishvili et al., 2019). Therefore, we first evaluated the BBB integrity 24 h following the induction of the stroke. Mice received a photothrombotic stroke with a size of 3 x 4 mm in the sensorimotor cortex and were systemically injected with Evans blue (EB), a BBB permeability marker 24 h before sacrifice (Figure 1A). EB has a high affinity for serum albumin, which does not cross the intact BBB to the brain parenchyma. 1 day following injury, stroked mouse brains exhibited a strong EB signal in the stroke core in the ipsilesional cortex and a lower signal in the surrounding peri-infarct zone (Figure 1B). The EB-positive core covered an area of 7.74 ± 3.52 mm^2^ with 11.55 ± 2.43 mm circumference, which was surrounded by an EB-positive peri-infarct region with a total area of 4.08 ± 1.46 mm^2^ (core + peri-infarct: 11.82 ± 4.98 mm^2^) and 13.756 ± 2.356 mm circumference. In the intact brain no signal of EB was detectable in any region (all *p* < 0.01, Figure 1C).

### Spatiotemporal Profile of Blood Brain Barrier Permeability Following Photothrombotic Stroke

The spatiotemporal profile of the BBB opening following human stroke is complex and may also occur beyond the acute stage (Merali et al., 2017; Nadareishvili et al., 2019). Re-openings of the BBB have been observed days to weeks following the initial ischemic injury in clinical and pre-clinical research (Huang et al., 1999; Kassner and Merali, 2015; Merali et al., 2017). Moreover, enhanced permeability has also been observed in regions not directly affected by the initial stroke due to retrograde or anterograde pathway degeneration and the associated inflammatory reaction (Ling et al., 2009; Li et al., 2011; Cao et al., 2020). Therefore, we aimed to characterize the spatiotemporal profile of BBB permeability up to 3 weeks following stroke.

**FIGURE 2:**
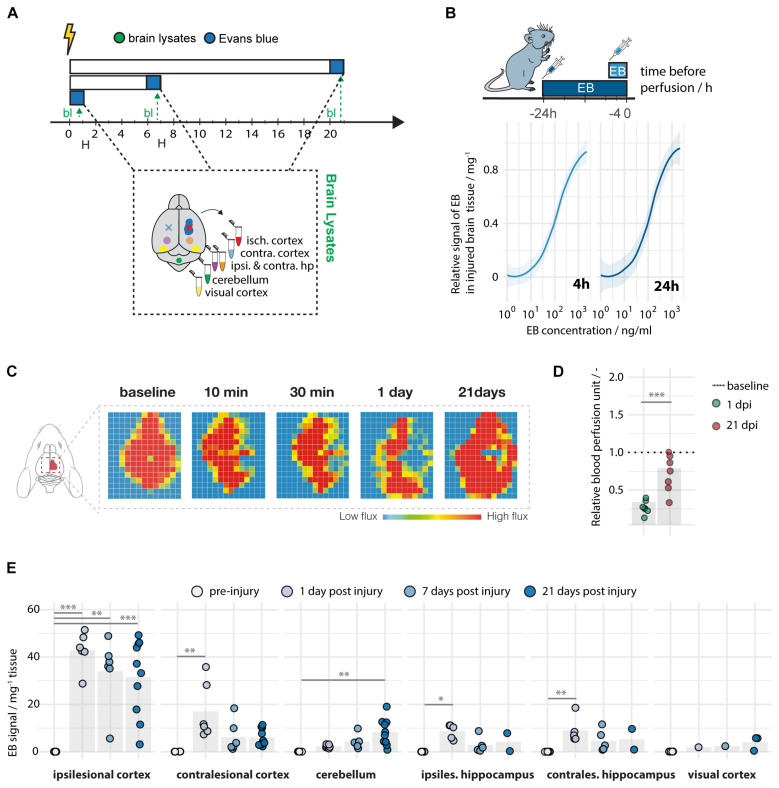
Spatiotemporal leakage following photothrombotic stroke. **(A)** Schematic representation of time course at baseline (*N* = 5), day 1 (*N* = 5–10), day 7 (*N* = 5–7), and day 21 (*N* = 2–10) following stroke. **(B)** Limit of detection and quantification of Evans blue in brain tissue. **(C)** Representative images of images of cerebral blood perfusion at 10, 30 min, 1 and 21 days following stroke. **(D)** Quantification of cerebral blood perfusion relative to baseline. **(E)** Quantitative assessment of Evans blue signal across different brain regions and time points. Data are represented as mean ± SD. Each dot in the plots represents one animal and significance of mean differences between the groups was assessed using Tukey’s HSD. Asterisks indicate significance: ^∗^*P* < 0.05, ^∗∗^*P* < 0.01, ^∗∗∗^*P* < 0.001 isch cx; ischemic cortex, contra cx; contralesional cortex, hp; hippocampus, cb; cerebellum, vcx, visual cortex. EB; Evans Blue, bl; brain lysates.

Brain tissue was dissected and lysed at 1, 7, and 21 days following a localized unilateral photothrombotic stroke to the sensory-motor cortex. Evans blue was detected at a high sensitivity with a limit of detection (LOD) of 0.006 ng and a limit of quantification (LOQ) of 0.019 ng per mg brain lysate, when administered either 4 or 24 h prior to perfusion (Figures 2A,B). A successful stroke procedure was confirmed by severe reduction in cerebral blood perfusion (CBF) of the sensorimotor cortex (<40% of baseline CBF) at 1 dpi (Figures 2C,D) using Laser Doppler Imaging (Figures 2C,D).

Interestingly, all mice showed up to 1,500-fold upregulation of the EB signal in the ipsilesional cortex 24 h following stroke [intact: 0.03 ± 0.01, stroke_(1 dpi)_: 42.91 ± 7.79] (Figure 2E). At later time points, most of the animals (83–90%) still exhibit enhanced leakage in the ischemic cortex [intact: 0.028, stroke_(7 dpi)_: 34.01 ± 14.77; stroke_(21 dpi)_: 31.54 ± 16.06, all *p* < 0.01]. Other brain regions, which were not directly affected by the initial stroke, also displayed enhanced although lower EB signals in individual animals. Most of these EB signals at 24 h after the stroke were detected in the contralesional cortex [intact: 0.05 ± 0.02, stroke_(1 dpi)_: 17.05 ± 11.92, *p* = 0.002], in the ipsilesional hippocampus [intact: 0.03 ± 0.01, stroke_(1 dpi)_: 8.98 ± 5.49, *p* = 0.03] and contralesional hippocampus [intact: 0.02 ± 0.01, stroke_(1 dpi)_: 8.63 ± 3.09, *p* = 0.002] and, at the latest time point, also in the cerebellum [intact: 0.06 ± 0.02, stroke_(21 dpi)_: 8.29 ± 5.59, *p* = 0.003] (Figure 2E). Importantly, signal intensities were not altered to acute time points before 24 h (Supplementary Figure S1).

**FIGURE 3:**
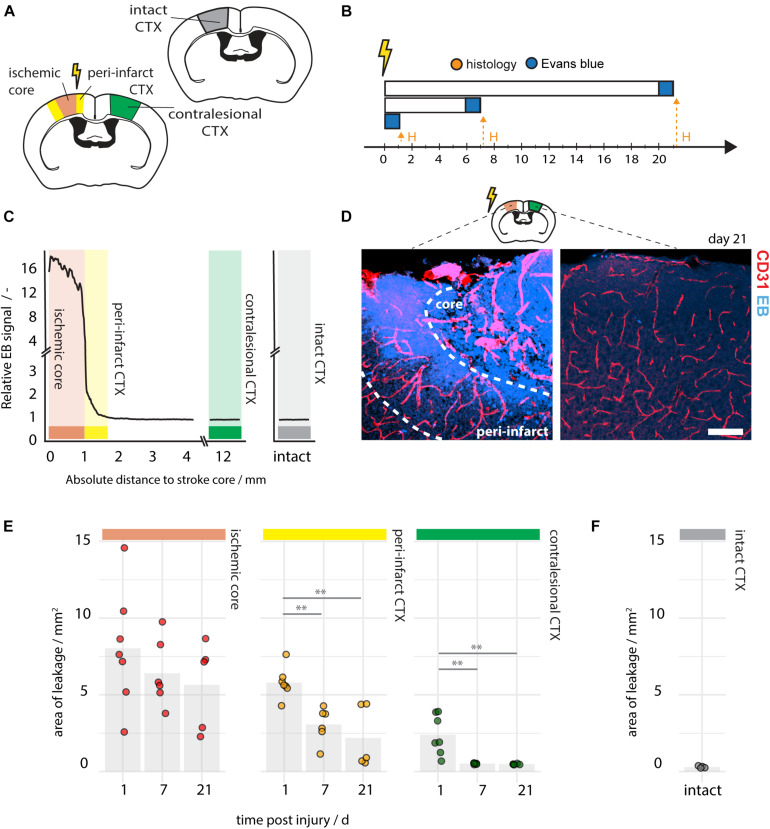
Histological assessment of BBB permeability following photothrombotic stroke. **(A)** Schematic representation of region of interest within the ischemic core (red), the peri-infarct region (yellow), the contralesional cortex (green), and the intact cortex (gray). **(B)** Time course of the experimental pipeline of 1 day (*N* = 8), 7 days (*N* = 6), and 21 days (*N* = 5) following injury. **(C)** Spatial distribution of EB signal along the ischemic, contralesional and intact cortex at 21 dpi. **(D)** Representative images of EB signal in the peri-infarct and contralesional region at 21 dpi. Scale bar: 100 μm. **(E)** Quantification of EB -positive area of the stroke core, the peri-infarct cortex and the contralesional cortex at days 1, 7, and 21 post injury. **(F)** EB -positive area in intact mice (*N* = 4). Each dot in the plots represents one animal and significance of mean differences between the groups was assessed using unpaired two-tailed one-sample Student’s *t*-test. Asterisks indicate significance: ^∗∗^*P* < 0.01. Dpi, days post injury; H, histology; ctx, cortex; EB, Evans blue.

In order to explore the spatial distribution of EB within the brain and to distinguish between core and peri-infarct leakage a histological analysis of vascular permeability in brain sections was performed on the ipsi- and contralesional cortex at day post injury 1, 7, and 21 (Figures 3A–C). EB signal strength was measured at the stroke core, the peri-infarct regions, in the contralesional cortex and in intact cortex by fluorescence intensity (Figure 3C). The area of EB leakage at the stroke core decreased slightly over time (1 dpi: 8.03 ± 3.83 mm^2^, 7 dpi: 6.40 ± 2.20 mm^2^, 21 dpi: 5.65 ± 2.80 mm^2^, all *p* > 0.05), whereas the EB -positive area in the peri-infarct regions steadily decreased from day 1 (5.79 ± 0.99 mm^2^) to day 7 (3.06 ± 1.13 mm^2^, *p* = 0.007), and day 21 (2.18 ± 2.01 mm^2^, *p* = 0.001) (Figures 3D,E). The peri-infarct region was defined by vascular density and ranged 300–600 μm from the stroke core as previously described (Rust et al., 2019b). On the contralesional side the area of EB leakage was 2.39 ± 1.30 mm^2^ at day 1 and shrunk to very small dimensions from day 7 on (Figure 3E). No EB signal was detectable in the cortex of intact mice (Figure 3F), nor the corpus callosum and other subcortical structures (data not shown).

In conclusion, most damage and vascular permeability in the photothrombotic stroke is present at the site of injury and it weakens gradually with distance to the stroke core. Non-affected brain regions can also show low to moderate leakage at different time points.

### Disruption of Peri-Vascular Cells and Junction Proteins Are a Feature of Photothrombotic Stroke

**FIGURE 4:**
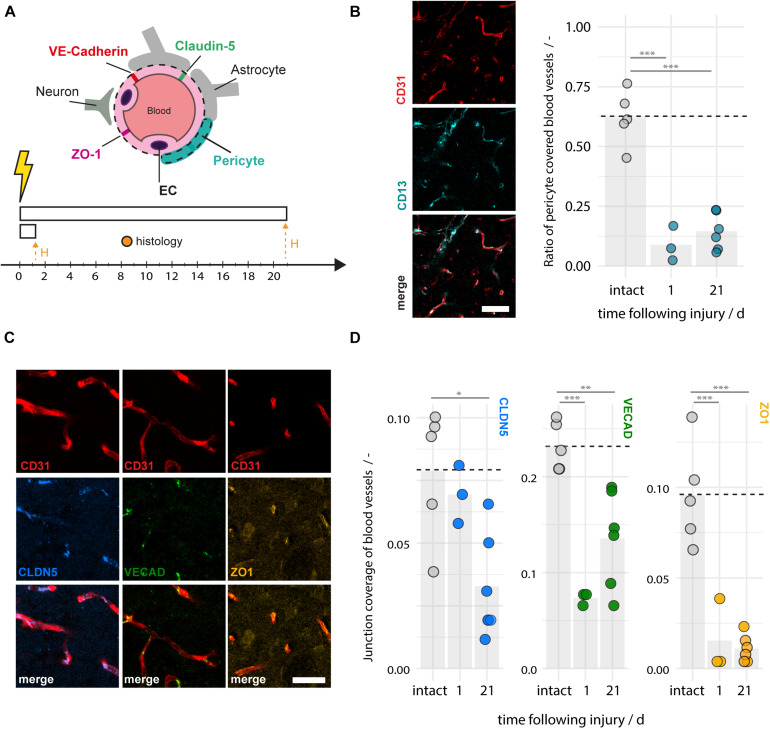
Disruption of pericyte coverage and tight and adherens junction proteins in the peri-infarct region 1–21 days after photothrombotic stroke. **(A)** Schematic representation of blood brain barrier composition and time course of experiment (*N* = 3–6). **(B)** Representative images of blood vessels (CD31, red) covered by pericytes (CD13, cyan) and the quantitative evaluation of pericyte coverage in intact and peri-infarct cortex at 1 and 21dpi. Scale bar: 50 μm. **(C)** Representative images of tight and adherens junction proteins Claudin5 (blue), VE-Cadherin (green), and ZO-1 (yellow) associated with blood vessels (CD31, red). Scale bar: 20 μm. **(D)** Quantitative evaluation of tight and adherens junction coverage of blood vessels in the intact and peri-infarct cortex at 1 and 21 dpi. Each dot in the plots represents one animal and significance of mean differences between the groups was assessed using unpaired two-tailed one-sample Student’s *t*-test. Asterisks indicate significance: ^∗^*P* < 0.05, ^∗∗^*P* < 0.01, ^∗∗∗^*P* < 0.001. CTX, cortex, H, histology.

The integrity of the BBB relies on tight and adherens junction protein complexes including Claudin-5, ZO-1, and VE-Cadherin (Jiao et al., 2011; Berndt et al., 2019) and cellular components of the neurovascular unit, especially pericytes (Armulik et al., 2010). Consequently, damage to any of these components may contribute to enhanced vascular leakage and the risk of hemorrhagic transformation. We histologically analyzed pericyte coverage and components of the junction proteins in the ischemic peri-infarct regions and intact animals at 1–21 days following photothrombotic stroke (Figure 4A). We observe a considerable ∼80% reduction of the pericyte fraction around blood vessels in the peri-infarct areas (1 dpi: 0.08 ± 0.07, 21 dpi: 0.15 ± 0.08, both *p* < 0.001) compared to intact cortex (0.62 ± 0.11) (Figure 4B). The surface ratio of blood vessels covered by tight/adherens junction components also significantly decreased in the peri-infarct region compared to the intact cortex. In particular, there was a decrease of 13% (1 dpi) and 59% (21 dpi) of Claudin-5 (1dpi: 0.069 ± 0.012, *p* = 0.832, 21 dpi: 0.033 ± 0.02 *p* = 0.013), a decrease of 68% (1 dpi) and 41% (21 dpi) of VE-Cadherin (1 dpi: 0.073 ± 0.006, *p* < 0.001; 21 dpi: 0.136 ± 0.05, *p* = 0.003), and a decrease of 85% (1 dpi) and 90% (21 dpi) of ZO-1 (1 dpi: 0.015 ± 0.020, *p* < 0.001; 21 dpi: 0.011 ± 0.007, *p* < 0.001) compared to the intact sensorimotor cortex (Claudin-5: 0.079 ± 0.026, VE-Cadherin: 0.232 ± 0.025, ZO1: 0.096 ± 0.023 (Figures 4C,D). Overall, these data indicated a considerable damage to the BBB anatomy also beyond the acute phase.

## Discussion

The blood brain barrier (BBB) has a central role in the pathogenesis of stroke. Disruption of the BBB occurs in the acute and subacute phases following stroke and is a precursor to serious clinical consequences such as brain edema and hemorrhagic transformation. Improving the BBB integrity following stroke has recently emerged as a focus for new therapeutic strategies (Sifat et al., 2017). Here, we characterize the spatiotemporal evolution of the BBB breakdown in a photothrombotic stroke model, a popular rodent model of ischemic stroke. While the BBB remained open in the stroke core, permeability decreased over the 3 weeks in the infarcted region. Anatomically, the enhanced permeability was correlated with a decrease of several major membrane constituents of the endothelial tight junctions, and with a reduction in the pericyte-to-blood vessel ratio. A subset of mice also exhibited low to moderate leakage in the contralesional cortex, the ipsi- and contralesional hippocampus and the cerebellum.

In stroke patients the disruption of the BBB was visualized *in vivo* by positron emission tomography (PET) (Okada et al., 2015), dynamic contrast-enhanced (DCE) magnetic resonance imaging (MRI) (Sourbron et al., 2009) and DCE computed tomography (CT) (Hom et al., 2011; Ozkul-Wermester et al., 2014). The dynamics of BBB permeability seem to vary somewhat between the studies. All reports show an acute opening of the BBB, and many report a bi-phasic or a continuous opening beyond the acute period with a maximum around day 2 and a second peak around day 7 (Merali et al., 2017). Similar results have been observed in animal models with well controlled experimental parameters. BBB openings have been observed in models of middle cerebral artery occlusion to follow bi-phasic course (Belayev et al., 1996; Rosenberg et al., 1998; Huang et al., 1999) or as a continuous opening (Nagel et al., 2004; Strbian et al., 2008).

Although various MCAO models of stroke are sufficiently documented with regard to BBB opening, there is insufficient knowledge in the time course of the photothrombotic stroke. Since the photothrombotic stroke is minimally invasive and reproducibly causes limb use deficits in mice for more than 16 weeks after the infarct, it is an interesting model for assessing long-term functional recovery (Allred et al., 2008). Consequently, efficacy of potential drugs interfering with BBB disruption can be evaluated using the photothrombotic stroke.

In our study, we observed a high variability within the experimental groups: Littermates undergoing the same surgical procedures and without obvious differences in stroke size showed considerable differences in BBB opening. In particular, leakage was clearly reduced in the peri-infarct region already at day 7 after the stroke, comparable to results from previous studies in a transient middle cerebral artery occlusion (MCAO) model (Strbian et al., 2008). However, some leakage, at least in part of the animals, was still detectable at 21 days in the peri-infarct zone. We found disruption of blood vessel pericytes coverage and tight junction components within the peri-infarct region at 1–21 days following stroke. Pericytes have been previously described to be an essential part of the BBB; pericyte-deficient mutant mice show significantly impaired BBB function (Armulik et al., 2010). Pericytes have also been described to be depleted in the peri-infarct region of MCAO strokes (Fernández-Klett et al., 2013; Nih et al., 2018). Although considerable sex differences have been previously reported in clinical stroke pathology (Reeves et al., 2008) as well as BBB permeability (Castellazzi et al., 2020), we have not observed any differences in BBB disruption between the sex in the photothrombotic stroke model.

Ischemia leads to the release of MMP9 by inflammatory cells in the peri-infarct region which interrupts the tight junctions and increases BBB leakage (Underly et al., 2017). Deficiencies in multiple tight junction proteins are a common observation in other ischemic rodent stroke models (Abdullahi et al., 2018). We have previously observed that sprouted, newly formed vessels in the peri-infarct region are initially immature and have less pericytes and tight junctions (Rust et al., 2019b,d). Therefore, leakage from these immature vessels may be an additional component of the BBB damage at the 7–21-day time points. However, most likely both events (damaged pre-existing vessels and newly formed immature vessels) contribute to the overall BBB disruption and it requires longitudinal *in vivo* experiments to identify the share of both processes. Moreover, since we performed end-point experiments in the study we have no information about levels of BBB damage outside of our measurements. Dissecting these mechanisms might be addressed in future studies with advancements in genetic models and two-photon microscopy.

An interesting observation in the photothrombotic model was the leakage in several brain regions remote from the stroke at different time points. This has also been observed in patients and other rodent stroke models and was mainly linked to degeneration and subsequent microglia activation and inflammation in projection areas or input systems to the stroke region with retrograde degeneration (Iizuka et al., 1990; Cao et al., 2017; Baumgartner et al., 2018; Jiang et al., 2018). mechanisms are especially known in the secondary thalamic injury affecting thalamus and hippocampus (Holmberg et al., 2009) but have been also observed in the cerebellum in our study. Further studies may address mechanistic basis for this effect in the photothrombotic or MCAO model.

Onset and duration of secondary injury-induced BBB leakage can vary between 3 days after stroke and may be detectable up to 6 months in rodents and 12 months in patients (Cao et al., 2020).

Taken together, the photothrombotic stroke model shows many features of BBB disruption that have been previously observed in stroke patients and other rodent stroke models. It therefore represents a suitable model to study BBB pathology and to develop therapies to improve BBB integrity following stroke.

## Materials and Methods

### Experimental Design

While BBB dysfunction has been implicated in stroke, the time-course of post-stroke BBB permeability changes is not well known for the photothrombotic stroke model. The present study aims to characterize the long-term temporal evolution of BBB opening following ischemic injury. We histologically and spectrophotometrically analyzed the loss of BBB integrity in different brain regions (affected and non-affected) at 1 days, 7, and 21 after photothrombotic stroke using Evans Blue (EB) dye to measure vascular leakage. To further investigate potential mechanisms underlying BBB damage, pericyte coverage and loss of major tight and adherens junction protein components in ischemic regions were quantified. For the time course experiment mortality rate was 0%. All animals are presented in the study; no statistical outliers were excluded. Data was acquired blinded.

### Animals

All animal experiments were performed in accordance with governmental, institutional (University of Zurich), and ARRIVE guidelines and had been approved by the Cantonal Veterinary Office of Zurich. Adult male and female wild type mice (10–14 weeks) of the C57BL/6 strain (16–25 g) were used. No sex-specific differences were observed in any of the experimental readouts. Mice were housed in standard Type II/III cages at least in pairs in a temperature and humidity controlled room with a constant 12/12 h light/dark cycle (light on from 6:00 a.m. until 6:00 p.m.).

### Surgical Procedure

Mice were anesthetized using isoflurane (3% induction, 1.5% maintenance; Attane, Provet AG). Analgesic (Novalgin, Sanofi) was administered 24 h prior to the start of the procedure via drinking water. A photothrombotic stroke to unilaterally lesion the sensorimotor cortex was induced on the right hemisphere, as previously described (Labat-gest and Tomasi, 2013; Rust et al., 2019b). Briefly, animals were placed in a stereotactic frame (David Kopf Instruments), the surgical area was sanitized and the skull was exposed through a midline skin incision. A cold light source (Olympus KL 1,500LCS, 150W, 3,000K) was positioned over the right forebrain cortex (anterior/posterior: −1.5–+1.5 mm and medial/lateral 0 –+2 mm relative to Bregma). 5 min prior to illumination, Rose Bengal (10 mg/ml, in 0.9% NaCl, Sigma) was injected intraperitoneally 5 min prior to illumination and the region of interest was subsequently illuminated through the intact skull for 8.5 min. To restrict the illuminated area, an opaque template with an opening of 3 × 4 mm was placed directly on the skull. The wound was closed using a 6/0 silk suture and animals were allowed to recover. For postoperative care, all animals received analgesics (Novalgin, Sanof) for at least 3 days after surgery.

### Blood Perfusion by Laser Doppler Imaging

The blood perfusion was measured using Laser Doppler Imaging (Moor Instruments, MOORLDI2-IR). Animals were placed in a stereotactic frame, the surgical area was sanitized and the skull was exposed through a midline skin incision. The brain was scanned using the repeat image measurement mode. All data were exported and quantified in terms of flux in the ROI using Fiji (ImageJ).

### Tissue Processing

To characterize the loss of BBB integrity after stroke, ischemic brain tissue was analyzed (1) histologically and (2) spectrophotometrically at day post injury 1, 7, and 21. EB, which was prepared as a 2% solution in saline, was injected intraperitoneally either 4 or 24 h prior to perfusion (6 μg/g body weight, Sigma). For histological analysis, animals were euthanized by intraperitoneal application of pentobarbital (150 mg/kg body weight, Streuli Pharma AG) and perfused with Ringer solution (containing 5 ml/l Heparin, B. Braun) followed by paraformaldehyde (PFA, 4%, in 0.2 M phosphate buffer, pH 7) to wash out intravascular EB. Brains were rapidly harvested and post-fixed for approximately 4 h by exposure to 4% PFA, then transferred to 30% sucrose for cryoprotection and stored at 4°C. Coronal sections with a thickness of 40 μm were cut using a sliding microtome (Microm HM430, Leica), collected and stored as free-floating sections in cryoprotectant solution at −20°C until further processing. For spectrophotometric analysis, animals were perfused with Ringer solution, CNS tissue was isolated (as described before) and stored at −20°C before further processing.

### Immunofluorescence

Brain sections were washed with 0.1M phosphate buffer (PB) and incubated with blocking solution containing donkey serum (10%) in PB for 30 min at room temperature. For detection of vascular endothelial cells, sections were incubated overnight at 4°C with monoclonal rat anti-CD31 antibody (BD Biosciences, 1:50). The localization of tight/adherens junction proteins was assessed using the following antibodies: mouse anti-Claudin-5 antibody (1:200, Thermo Fisher Scientific); rat anti-VE-Cadherin antibody (1:100, Thermo Fisher Scientific), and rabbit anti-ZO-1 antibody (1:100, Thermo Fisher Scientific). Pericyte coverage was visualized with goat anti-CD13 (1:200; R&D Systems). The primary antibody incubation was followed by 2 h incubation at room temperature with corresponding fluorescent secondary antibodies (1:500, Thermo Fisher Scientific). Nuclei were counterstained with DAPI (1:2,000 in 0.1 M PB, Sigma). Sections were mounted in 0.1 M PB on Superfrost PlusTM microscope slides and coverslipped using Mowiol.

### Spectrophotometric Evans Blue Quantification

Evans Blue dye is an inert tracer frequently used in BBB studies. EB binds rapidly and exclusively to plasma albumin (Yen et al., 2013) when injected peripherally into circulation. Since serum albumin does not cross the BBB to the brain parenchyma if the barrier is structurally and functionally intact, spectrophotometric determination of EB dye accumulation in brain tissue outside blood vessels reflects the extent of vascular leakage. The isolated CNS tissue samples were homogenized in a lysis buffer (250 μl/mg tissue weight; Tris-HCl, EDTA, NP-40, NaCl, protease inhibitor cocktail) and incubated for 2 h at 4°C, shacking. The mixture was centrifuged to sediment the non-dissolved tissue parts (25 min, 15,000 g, 4°C) and the extracted supernatant was collected in a 96-well plate. Based on a standard curve, EB concentrations could be quantified from their absorbance readings (620 nm) using a standard microplate reader (Spark, Tecan). The results were expressed as micrograms of EB per milligram of brain tissue.

### Fluorescence Microscopy and Quantification

Imaging of brain sections was performed 1, 7, and 21 days after stroke with an Olympus FV1000 or Leica SP8 laser scanning confocal microscope equipped with 10×, 20×, and 40× objectives. Images were processed using Fiji (ImageJ), Adobe Photoshop CC and Adobe Illustrator CC. Evans blue signal area in ischemic brain sections was quantified by thresholding at fivefold background signal intensity of the intact sensorimotor cortex (perfused with EB). Pericyte and tight junction coverage of blood vessels was calculated as previously described with an automated Fiji (ImageJ) script (Rust et al., 2019a, 2020) and normalized to the intact cortex. Briefly, 4–6 random ROIs were selected. Area covered by blood vessels was enlarged by 3 μm and pericyte or tight junction signals within this mask were binarized and presented as a ratio. We defined the stroke core region as the region with no surviving neurons that is surrounding by the glial scar. From this region, we defined tissue up to 300 μm distal along the cortex as the ischemic border zone as previously described (Rust et al., 2019b).

### Statistical Analysis

Statistical analysis was performed using RStudio and GraphPad Prism 7. Sample sizes were designed with adequate power according to our previous studies (Rust et al., 2019b,d) and to the literature. All data were tested for normal distribution by using the Shapiro-Wilk test. Normally distributed data were tested for differences with a two-tailed unpaired one-sample *t*-test to compare changes between two groups (differences between ipsi- and contra-lesional site) as in Figures 3E, 4B–D. Multiple comparisons as in Figures 1C, 2D were initially tested for normal distribution with the Shapiro-Wilk test. The significance of mean differences between normally distributed multiple comparisons was assessed using Tukey’s HSD. Data are expressed as means ± SD, and statistical significance was defined as ^∗^*p* < 0.05, ^∗∗^*p* < 0.01, and ^∗∗∗^*p* < 0.001.

## Data Availability Statement

The raw data supporting the conclusions of this article will be made available by the authors, without undue reservation.

## Ethics Statement

The animal study was reviewed and approved by the Cantonal Veterinary Office of Zurich.

## Author Contributions

RW, LG, and RR designed the study. RR prepared the figures and wrote the manuscript. RW, LG, GM, MM, and RR carried out the experiments. RW, LG, MS, and CT proofread and revised the manuscript. All authors read and approved the final manuscript.

## Conflict of Interest

The authors declare that the research was conducted in the absence of any commercial or financial relationships that could be construed as a potential conflict of interest.
