# Supplementary material for: Characterization of the Blood Brain Barrier Disruption in the Photothrombotic Stroke Model
Source: Front Physiol. 2020 Nov 12;11:586226. doi: 10.3389/fphys.2020.586226 (PMC7688466; doi:10.3389/fphys.2020.586226)
Supplement: Supplementary file 1 [file Data_Sheet_1.PDF]

Supplementary Material

Supplementary Material

A

B

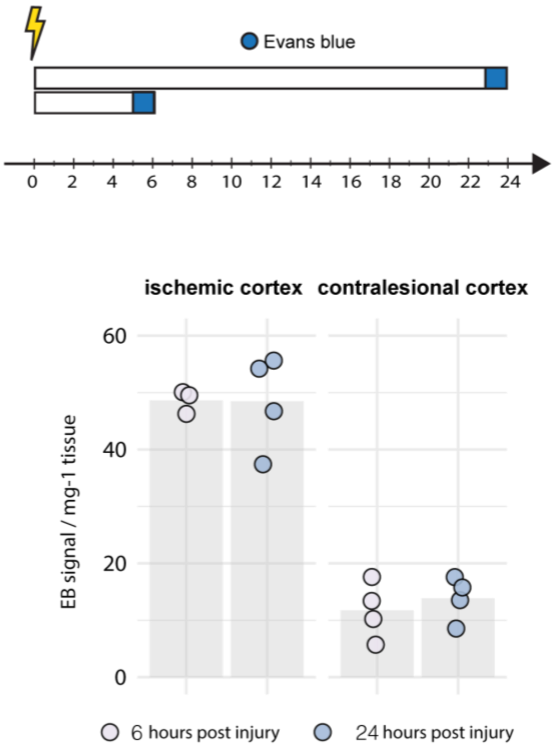

Supplementary Figure 1: Acute effects of BBB leakage following photothrombotic stroke. Time course at 6 hours (N=4) and 24 hours (N=4) following stroke. B) Quantification of EB signal in lysed brain parenchyma at 6 and 24 hours following injury in the ischemic and contralesional cortex. Each dot in the plots represents one animal and significance of mean differences between the groups was assessed using unpaired two-tailed one-sample Student's t-test.
